# Lateral Abdominal Muscles Shear Modulus and Thickness Measurements under Controlled Ultrasound Probe Compression by External Force Sensor: A Comparison and Reliability Study

**DOI:** 10.3390/s21124036

**Published:** 2021-06-11

**Authors:** Grzegorz Mikołajowski, Małgorzata Pałac, Tomasz Wolny, Paweł Linek

**Affiliations:** 1 Musculoskeletal Elastography and Ultrasonography Laboratory, Institute of Physiotherapy and Health Sciences, The Jerzy Kukuczka Academy of Physical Education, 40-065 Katowice, Poland; g.mikolajowski@awf.katowice.pl (G.M.); malgorzatapalac3@gmail.com (M.P.); t.wolny@awf.katowice.pl (T.W.); 2 Musculoskeletal Diagnostic and Physiotherapy—Research Team, Institute of Physiotherapy and Health Sciences, The Jerzy Kukuczka Academy of Physical Education, 40-065 Katowice, Poland

**Keywords:** elasticity, ultrasound imaging, adolescence, abdominal muscles, reliability, validity, reproducibility, shear modulus, thickness

## Abstract

The aim of this study was to perform a reliability and agreement analysis and to compare lateral abdominal muscles (LAMs) thickness and elasticity results obtained by an experienced operator, by a non-experienced operator, and in an ultrasound imaging probe compression controlled (PCC) condition with minimal force necessary to obtain a proper ultrasound image. The sample consisted of 39 adolescents. An Aixplorer ultrasound scanner was used to evaluate the LAM. The probe in PCC condition was positioned in a prepared probe holder coupled with a pressure sensor. The LAM thickness and elasticity measurements were significantly (*p* < 0.05) different in the ultrasound PCC condition, compared to results obtained by both examiners. The abdominal oblique external and internal muscle thickness measurements were underestimated and all LAM shear moduli were overestimated during measurements without controlling the probe compression by an external sensor. The intra-class correlation coefficient was excellent in all conditions, but the smallest detectable differences were approximately 43–60% lower during the measurements collected in PCC condition. Differences in LAM measurements between PCC and ‘on-hand’ conditions may be clinically irrelevant when the force applied by the probe is consciously controlled by the examiner. However, during ultrasound measurements of the LAM morphology, the potential under/over estimation should always be considered when measurements are performed without controlling probe compression by an external sensor.

## 1. Introduction

For the last few decades, B-mode ultrasound imaging (USI) has been used to assess muscles’ geometry. In some cases, USI has been used to assess lateral abdominal muscles (LAMs) thickness or thickness change at rest and during various movements because the potential role of LAM in low back pain (LBP) [1,2,3] and scoliosis [4,5,6,7,8,9,10,11] was verified. Recently, quantitative shear wave ultrasound elastography (SWE) has also been developed and used to study muscle shear modulus [12] and refer it to muscles’ elasticity. Thus, LAMs geometry (thickness or thickness change) and elasticity are routinely measured in scientific research using ultrasonography.

Numerous studies have analyzed the reliability of LAMs thickness measured by USI, taking into account various health conditions (LBP, scoliosis, and healthy participants), movement tasks, body positions, respiratory cycle phase, participants’ age, and operator’s experience [13,14,15,16,17,18,19]. A recent systematic review showed that USI has good inter- and intra-rater reliability, and the reported standard error of measurement and minimal detectable change for USI were in an acceptable range [20]. To date, the reliability of LAMs shear modulus measurements by SWE were conducted in three studies [16,21,22]. The reliability results on adults have shown fair to excellent intra- and inter-rater reliability [21]. However, data from some participants revealed that the transversus abdominis muscle’s (TrA) elasticity showed poor reliability under some conditions [21]. In adolescents, in turn, the SWE reliability and agreement was appropriate for all LAMs [16]. Thus, it was confirmed that SWE for LAMs shear modulus measurements has greater potential for use in the pediatric population because the level of reliability and agreement during shear modulus measurements depended on the muscle’s location (particularly depth) [16]. Muscles located more superficially are more easily analyzed by SWE. In the pediatric population, the muscles and the superficial fat layer are thinner than in adults [16,23,24]. This explains the better utility of SWE in the pediatric population and may be used to assess passive and active muscle force or fatigue in some musculoskeletal conditions. However, the more superficially located muscles of the pediatric population may cause higher (compared to adults) measurement error due to probe compression.

The European Federation of Societies for Ultrasound in Medicine and Biology (EFSUMB) has recommended minimizing probe pressure during the imaging of superficial organs [25]. However, the abdominal area and region to assess LAMs is curved. Such a curved area, compared to a flat area, is more challenging to image by USI without probe compression. Some researchers have stated that it is impossible to obtain a proper USI without applying some pressure to the measured tissue due to the curved body surface [26]. They have suggested that the USI and SWE results are affected by tissue compression [26,27,28]. Linek et al. [16] suggested that the force applied by the probe may have a negative effect on the reliability and agreement of the LAMs elasticity and thickness measurements performed by USI, so the force should be consciously controlled by the operator. Unfortunately, conscious control of the ultrasound probe compression does not guarantee that the obtained results are true, and the real deviation from the results gained while controlling the force applied are not known. To the best of our knowledge, there was no study quantifying the extent to which the results of LAMs thickness and elasticity differ between a manually operated probe and a probe with controlled compression by an external force sensor. Such information seems to be important in scientific research when comparing different health conditions or developing a reference database.

In some USI reliability studies, the aspect of operator experience was considered. It is accepted that an unskilled operator (after training) is able to obtain an appropriate level of reliability without systematic errors [21,29]. However, there were situations in which less experienced operators obtained less thick muscle and a higher mean shear modulus, compared to more skilled operators [16,29]. This means that the force applied by unexperienced operators was probably too high during USI. The existing LAMs thickness and elasticity reliability studies have not controlled probe compression force: usually, only the general statement that an ‘excessive amount of transduction gel was used to reduce the load applied to the skin’ has been applied. Thus, based on the present knowledge, we still do not know the measurement deviations between skilled operator and optimal condition (no force or minimal force between probe and skin) when assessing LAMs shear modulus and thickness by USI. Therefore, the aim of this study was to (i) compare LAMs thickness and elasticity results obtained by an experienced operator, by a non-experienced operator, and with probe compression controlled (PCC); (ii) present a reliability and agreement analysis for each of these conditions.

## 2. Materials and Methods

### 2.1. Study Design

A prospective single-blinded test/retest design was used to compare differences of the remaining LAMs elasticity and thickness values between experienced/non-experienced examiners and results gained by PCC. The order of measurements and body side was randomized prior to the study. The study was conducted in accordance with the Declaration of Helsinki, and the protocol was approved by the Ethics Committee of the Jerzy Kukuczka Academy of Physical Education in Katowice (4/2017).

### 2.2. Participants

The convenient sample consisted of 39 (mean age: 11.6 ± 1.76 years; mean body mass: 41.8 ± 10.7 kg; mean body height: 150.9 ± 13.8 cm; BMI: 18.1 ± 2.77 kg/m^2^) children and adolescents. Participants eligible for inclusion were children and adolescents (younger than 18 years). The exclusion criterion were: (a) scoliosis confirmed on X-ray imaging; (b) had experienced LBP during the day of examination; (c) any prior surgery on the abdominal and/or spinal regions; (d) the parents were unwilling to provide written informed consent. All participants gave their informed consent for inclusion before they participated in the study.

### 2.3. Investigators

SWE and thickness measurements were performed by three independent physiotherapists. Prior to the study, examiner A had 12 years of experience in musculoskeletal ultrasound. Examiner B underwent 30 h of theoretical and practical training in assessing LAMs thickness and elasticity, and had 3 months of experience in assessing LAMs in the pediatric population. Examiners A and B were blinded to the study aim. Measurements with technically controlled ultrasound probe compression (PCC condition) were performed by examiner C (9 years of experience in musculoskeletal ultrasound; the most experience in LAMs scanning).

### 2.4. Equipment and Data Analysis

In order to assess LAMs elasticity (shear modulus) and thickness, an Aixplorer ultrasound scanner (Supersonic Imagine, Aix-en-Provence, France) with a linear transducer array (2–10 MHz; SuperLinear 10-2, Vermon, Tours, France) has been used in the SWE mode. This mode is dedicated to elasticity measurements. All images were stored in the ultrasound scanner and calculated after collecting data from all participants. The thickness of the superficial fat layer was additionally measured in the thickest part of each image. The transducer was always positioned on the anterolateral wall, at the level of the umbilicus and parallel to the umbilicus. The transducer was orientated perpendicular to the long axis of the body (along the line of muscle fibers of TrA). Before the image was stored, the probe was adjusted to visualize the TrA myofascial junction. In further analysis, muscle and fat layer thickness from two images were used.

**Figure 1:**
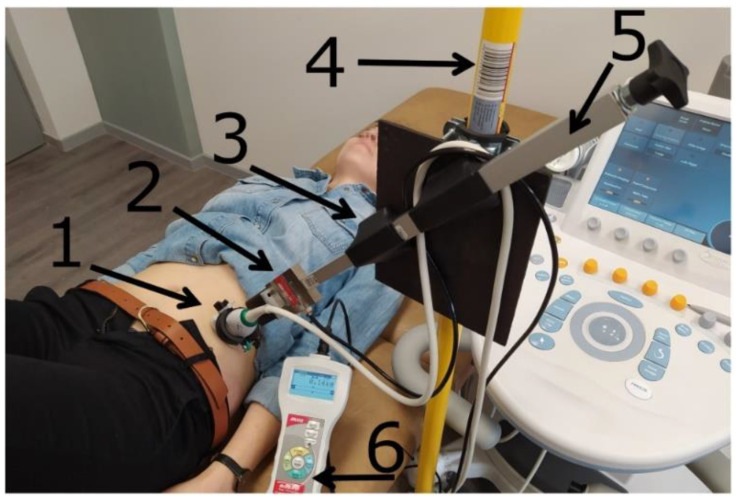
General overview of the setup for the ultrasound examination with probe compression controlled. 1. Ultrasound probe. 2. External S-Type load cell connected to a force gauge (6). 3. Two-dimensional magnetic holder. 4. An expansion beam. 5. Precise screw actuator.

Examiners A and B performed measurements without any additional tools, whereas examiner C used a probe holder, specially prepared by the first author (GM), coupled with a pressure sensor. The pressure sensor (external S-Type load cell—DEE, Keli Sensing technology, Ningbo, China) was coupled with a force gauge FB1k (Axis, Gdansk, Poland). The force gauge was connected via a USB port to a computer and controlled by the AXIS FM v2.09 program (AXIS, Gdansk, Poland). This allowed the display of the force value (in Newtons) on screens in real time (Figure 1). The probe holder with the sensor was placed on an expansion beam by a magnetic holder. It allowed adjusting the probe in two dimensions without any restrictions. The probe was moved toward the subject’s body with a screw, and the compression force was registered by the pressure sensor and depicted on the force gauge.

### 2.5. Measurement Procedures

The measurements were collected in the resting semi-supine position. The participants were asked to breathe quietly, and the SWE image was taken at the end of normal expiration. Each participant was familiarized with the procedure by a technical assistant who was aware of the study aim and all procedures. The technical assistant asked participants to breathe quietly, stay calm, and not move on the therapeutic couch. Then, examiner A, B, or C (in randomized order) was invited to measure the shear modulus and thickness of the LAMs. The body side (right or left) of the measurements was also randomized. Each examiner collected two separate images only on one body side (right or left).

Before the study, examiners A and B were encouraged to obtain measurements following general rules during ultrasound examination. Both researchers were reminded of the necessity to apply minimum force by probe to the skin. Examiner C used the probe holder to adjust the probe in order to get a proper ultrasound image. The probe was moved toward the subject’s body with a screw until the proper image was obtained. The compression force was controlled by external S-Type load cell and depicted on the force gauge. Examiner C was obligated to get proper USI without or with minimal (as few as possible) detected compression force. All examiners used an excessive amount of hypoallergenic transduction gel to reduce the potential load applied to the skin. Additionally, all examiners were reminded to position the probe in the proper place (at the level of the umbilicus, parallel to the umbilicus, perpendicular to the long axis of the body) and adjust the probe to produce the closest to optimal image. During the scanning procedure performed by the first examiner, the technical assistant marked the internal and upper border of the probe on the participant’s skin to allow other examiners find the same placement for their scanning.

**Figure 2:**
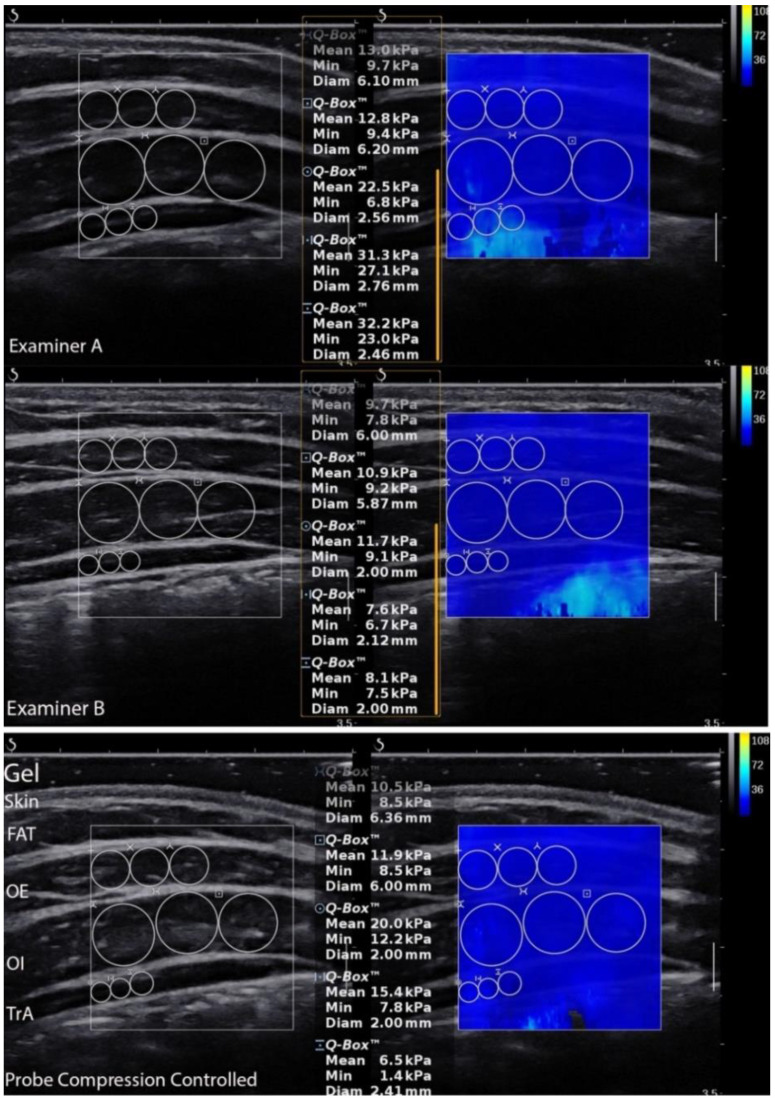
Ultrasound scans of the lateral abdominal muscles performed by all examiners and shear modulus measurements. FAT—superficial fat layer; OE—oblique external; OI—oblique internal; TrA—transversus abdominis muscle.

**Figure 3:**
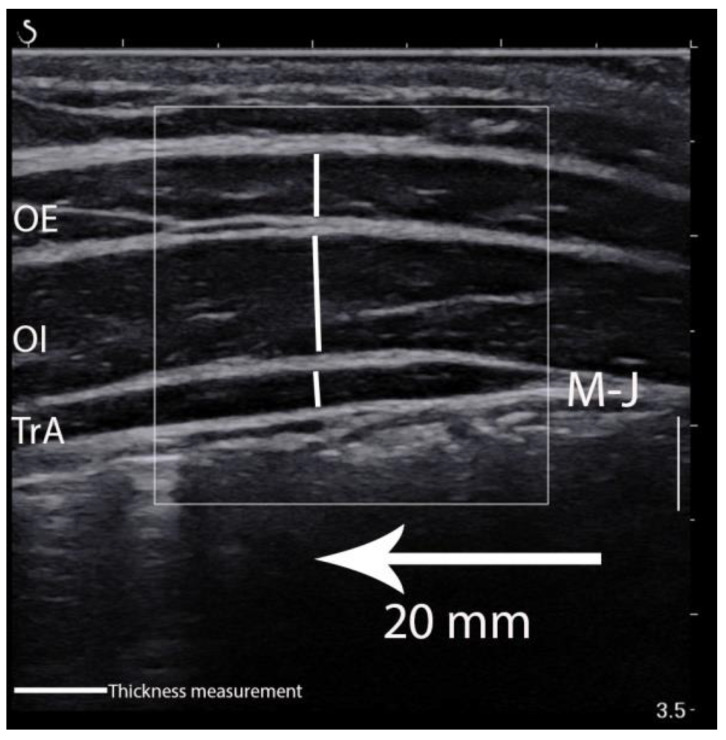
Thickness measurement of the lateral abdominal muscles. OE—oblique external; OI—oblique internal; TrA—transversus abdominis muscle; M-J—myofascial junction of the TrA.

LAMs elasticity was calculated in the ultrasound scanner using the Q-Box quantitative tool after collecting all data (Figure 2). This was used to quantify each muscle shear modulus. Three separate circles were positioned inside the fascial edge of each muscle, and the muscle elasticity within the circle was automatically calculated and depicted in kilopascal (kPa). The mean value of the three separate circles was considered the muscle elasticity value from a given image [16]. In turn, images for LAMs thickness and superficial fat layer measurements were edited and calculated using RadiAnt Dicom Viewer 5.5.1 (Medixant, Poznań, Poland). The edits included enlarging, brightening, and adding contrast. The vertical marking line positioned 20 mm from the musculofascial junction of the TrA was consistently used to re-measure the thickness for each muscle (Figure 3). The vertical distance between the musculofascial layers represented the individual thickness of the oblique external (OE), oblique internal (OI), and TrA [24,30].

### 2.6. Statistical Analyses

To calculate reliability, the intra-class correlation coefficient (ICC—model 3,1) was used. The ICC was categorized as follows: 1.00–0.75 (excellent), 0.74–0.60 (moderate), 0.59–0.40 (fair), and below 0.40 (poor reliability) [31]. For the calculation of agreement, the smallest detectable differences $\left(\mathrm{SDD}=1.96\times \;\mathrm{SEM}\;\times \sqrt{2}\right)$ and the results of the Bland–Altman test (BA) were used. The BA test was only used to find potential biases between the two measures. Taking into account that the sample size was not large enough (preferably greater than 50) to allow the limits of agreement to be accurately estimated [32], plots with limits of agreement were not included.

Normal distribution was checked by the Shapiro-Wilk test. As it was in violation of this assumption, a logarithmic transformation was successfully performed to obtain normal distribution. The log transformed data were analyzed with one-way ANOVA for repeated measurements, with no between-subject factor and within factor being LAMs results from all examiners (results from examiner A vs. examiner B vs. examiner C). For significant main effect, a Bonferroni post hoc analysis was performed. The results are presented as a mean difference and 95% confidence interval (CI). The significance level was set at *p* < 0.05. Data were analyzed using STATISTICA 13.1 PL (Statsoft, Tulsa, OK, USA) and Excel (Microsoft Corporation) software.

## 3. Results

### 3.1. Reliability

The intra-examiner ICC for thickness measurements with PCC was excellent (all ICC > 0.90), without any systematic error examined in the BA test. Examiners A and B received slightly worse but still excellent ICC for OE, OI, and superficial fat layer with no systematic errors. Unlike measurements with PCC, examiners A and B received similar systematic errors examined in the BA test: examiners A and B recorded a thicker TrA muscle during the second measurement. The corresponding SDD values were about 43–60% lower during the measurements collected with PCC, compared to examiners A and B (Table 1).

The intra-examiner ICC for LAMs elasticity measurements ranged from fair to excellent (0.55–0.97). There was no systematic error examined in the BA test for PCC and examiners A and B. The most notable differences were for SDD values for OE and OI; for measurements performed with PCC, the SDD was 2–3 kPa, whereas for measurements performed by examiners A and B, the SDD was 5.5–7 kPa (Table 1).

### 3.2. Between-Examiner Differences

**Figure 4:**
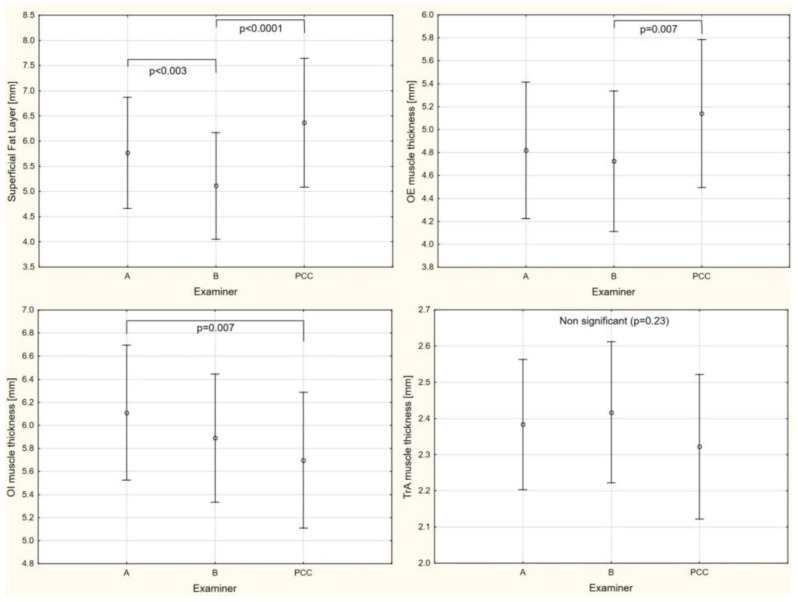
Absolute muscle and superficial fat thickness performed by examiner A, B, and with probe compression controlled (PCC). OE: Oblique external; OI: Oblique internal; TrA: Transversus abdominis.

For superficial fat, OE, and OI muscle thickness, a significant effect of examiners was demonstrated (*p* < 0.05). A post hoc analysis showed that examiner C (PCC condition) had a thicker superficial fat layer by 1.25 mm (95% CI 0.85–1.66) and OE muscle by 0.41 mm (95% CI 0.12–0.71) than examiner B. Additionally, examiner A had a significantly thicker superficial fat layer by 0.66 mm (95% CI 0.25–1.06) than examiner B. For IO muscle thickness, a post hoc analysis showed that examiner C had a thinner OI muscle by 0.41 mm (95% CI 0.08–0.74) than examiner A. With regard to the TrA muscle, a significant main effect of examiners was not demonstrated (Figure 4).

**Figure 5:**
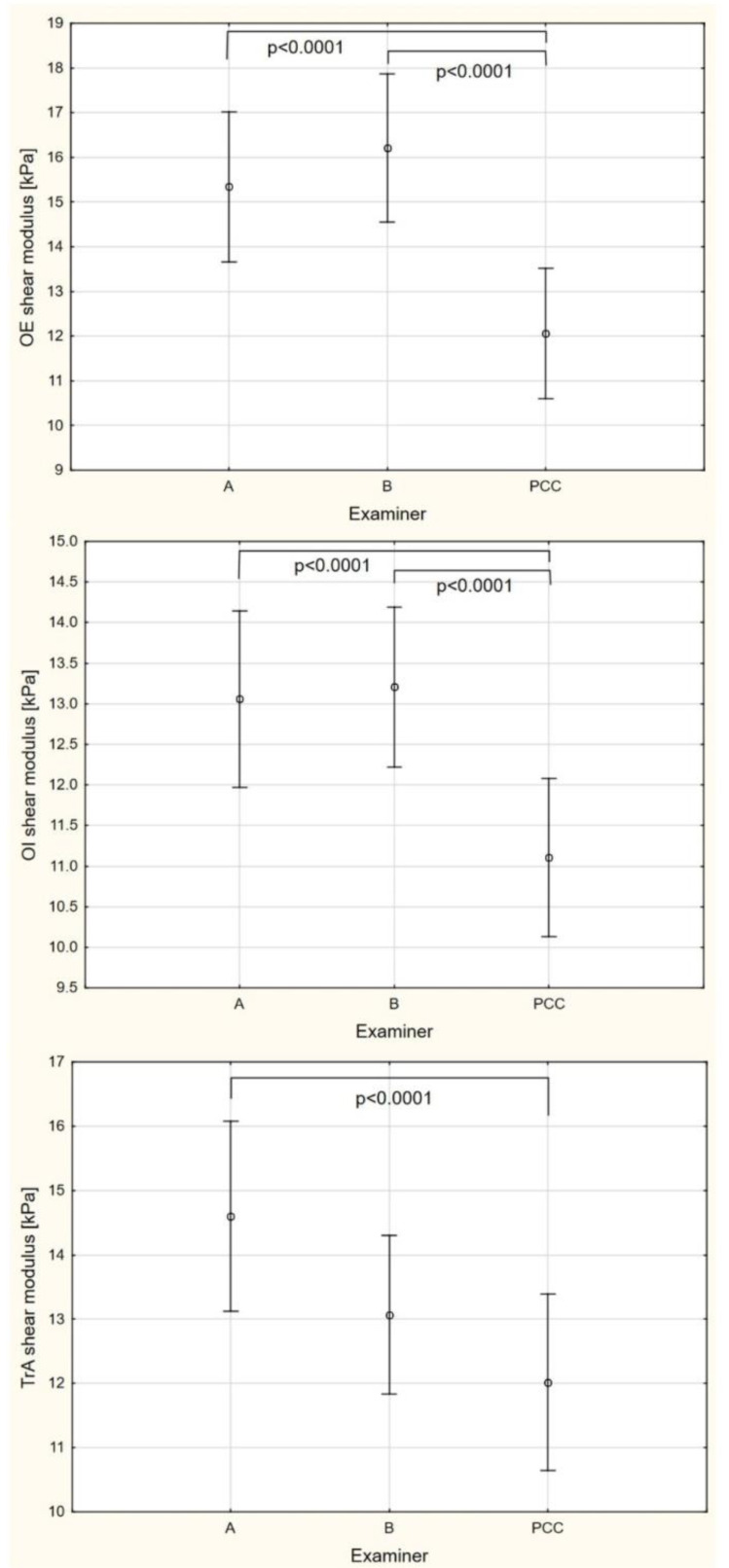
Absolute muscle elasticity performed by examiner A, B, and with probe compression controlled (PCC). OE: Oblique external; OI: Oblique internal; TrA: Transversus abdominis.

For OE, OI, and TrA muscle elasticity, a significant effect of examiners was demonstrated (*p* < 0.05). A post hoc analysis showed that examiner C had a lower OE muscle stiffness by 3.29 kPa (95% CI 2.12–4.46) and 4.15 kPa (95% CI 2.98–5.32) than examiners A and B, respectively. Similarly, for OI muscle elasticity, examiner C had a lower OI muscle stiffness by 1.95 kPa (95% CI 0.87–3.04) and 2.10 kPa (95% CI 1.01–3.19) than examiners A and B, respectively. For TrA muscle elasticity, a post hoc analysis showed that examiner C had a lower TrA muscle stiffness by 2.58 kPa (95% CI 1.04–4.13) than examiner A (Figure 5).

## 4. Discussion

The primary aim of the study was to compare LAMs thickness and elasticity measurements obtained by an experienced operator, by a non-experienced operator, and in the ultrasound PCC condition. Additionally, the second study aim was to assess the reliability and agreement of LAMs shear modulus and thickness measurements performed by experienced and non-experienced examiners as well as by ultrasound PCC. Our study has shown that the reliability of LAMs thickness measurements is weakly affected by ultrasound probe compression performed by examiners A and B, but the USI and SWE examination should follow the EFSUMB recommendation (minimal probe pressure). However, the agreement for LAMs thickness was generally worse for examiners A and B compared to the PCC condition, and systematic error for the TrA thickness measurement performed by examiner A and B was detected. With regard to LAMs shear modulus measurements, the reliability in the PCC condition was the highest, compared to examiners A and B. The SDD in the PCC condition was twice as low as for examiners A and B. This study has also shown that the LAMs thickness and elasticity measurements were different in the ultrasound PCC condition, compared to results obtained by experienced and non-experienced examiners. All these differences were obtained despite the explicit suggestion to use minimum probe compression during the examination performed by examiners A and B. Fortunately, the difference between examiners A and B, compared to the PCC condition, seems to be clinically irrelevant, because the SDD value is similar or sometimes higher than the mean difference.

To the best of our knowledge, no other studies have assessed the extent to which the results of LAMs thickness and elasticity depend on the pressure applied to the probe. This study has confirmed other authors’ suggestions [26,27,28] that the geometry (thickness) and elasticity results are affected by tissue compression. Vachutka et al. [26] stated that it is difficult to obtain proper USI without applying pressure to the measured tissue due to the curved body surface. The abdominal area is rounded, and ultrasound LAMs thickness or elasticity measurements need some compression force applied by the probe. Linek et al. [16] suggested that the ultrasound probe should be consciously controlled by the operator, because the force applied by the probe may have a negative effect on LAMs elasticity and thickness measurements. Our study confirmed this, despite the fact that the examiners were encouraged to pay attention to the probe compression. The results obtained in the PCC condition were significantly different from those obtained by examiners A and B. This means that, when holding the ultrasound probe in the examiner’s own hand, a higher level of probe compression should be expected, compared to an external probe holder.

Other studies have shown that unskilled examiners (after training) are able to obtain an appropriate level of reliability without any systematic errors during LAMs shear modulus measurements [21,29], but less experienced examiners obtain less thick muscle and higher mean shear wave speed, compared to more skilled examiners [16,29]. This was also confirmed in our study. Thus, during ultrasound training of LAMs, the aspect of probe compression should be highlighted as a potential source of measurement errors.

Some limitations should also be taken into account in this research. First, the study was conducted on an adolescent population. There is a high probability that the results will be different in an adult population due to different (higher) muscle thickness and superficial fat content in adulthood. It was suggested that in some situations the superficial fat layer may work as a ‘pillow’ suppressing and distributing the force applied by the probe [16]. This means that thickness and elasticity measurements may be less affected by probe compression in people with a thicker superficial fat layer. However, the acoustic velocity of fat in the body is approximately 100 m/s higher than that of muscle [33,34]. This is an another potential source of error and unreliability during shear modulus assessments [35,36] because subcutaneous fat attenuates propagation of the external stimulus applied at the skin surface [37]. Second, prior to the study, examiners A and B were encouraged to pay more attention to probe compression while performing measurements. Thus, it could be possible that, in clinical practice, the disproportion between on-hand USI and PCC will be higher. Third, although the probe position was standardized during the scanning procedure, the examiners were not controlled in this regard. Thus, some minimal disproportions may have occurred.

## 5. Conclusions

Ultrasonography measurements of LAMs thickness and shear modulus differ between manually operated probes and probes with controlled compression by external force sensor. Superficial fat layer and OE and OI thickness measurements were underestimated and all LAMs shear modulus were overestimated during measurements without controlling the probe compression by an external sensor. Regardless of examiners, the ICC was excellent, but the SDD values were about 43–60% lower during the measurements collected with controlled probe compression. Thus, differences in LAMs measurements between PCC and ‘on-hand’ conditions may be clinically irrelevant when the force applied by the probe is consciously controlled by the examiner. However, during USI of the LAMs, the potential under/over estimation should always be considered when measurements are performed without controlling probe compression by an external sensor. In laboratory situations where maximum precision of LAMs measurement is required, the use of a probe holder is recommended instead of taking measurements ’on-hand’ (even if performed by an experienced researcher).

## Figures and Tables

**Table 1:** Reliability and validity of elasticity and thickness values of oblique external (OE), oblique internal (OI), and transversus abdominis (TrA) muscles in supine rest position.

			OE	OI	TrA	Fat
Shear Modulus (kPa)	Intra-rater Reliability Rater A	ICC_3.1_	0.83	0.71	0.70	
		SDD (kPa)	6.16	5.41	7.49	
		Bias ^2^ (kPa)	−0.96	−0.46	−0.12	
	Intra-rater reliability Rater B	ICC_3.1_	0.78	0.63	0.55	
		SDD (kPa)	6.99	5.64	8.00	
		Bias ^2^ (kPa)	0.27	0.23	−0.21	
	Intra-rater reliability Probe compression controlled	ICC_3.1_	0.97	0.88	0.73	
		SDD (kPa)	2.17	2.96	6.57	
		Bias ^2^ (kPa)	0.21	−0.40	−0.88	
Muscle or Fat Thickness (mm)	Intra-session reliability Rater A	ICC_3.1_	0.93	0.91	0.81	0.96
		SDD (mm)	1.36	1.52	0.71	1.90
		Bias ^2^ (mm)	0.09	0.09	−0.17 ^1^	−0.01
	Intra-session reliability Rater B	ICC_3.1_	0.92	0.88	0.70	0.93
		SDD (mm)	1.50	1.69	0.99	2.42
		Bias ^2^ (mm)	0.17	−0.07	−0.18 ^1^	0.07
	Intra-rater reliability Probe compression controlled	ICC_3.1_	0.99	0.97	0.93	0.99
		SDD (mm)	0.55	0.90	0.46	1.09
		Bias ^2^ (mm)	−0.03	−0.07	0.01	0.04

^1^ systematic error as the line of equality is not in the 95% confidence interval; ^2^ Bland–Altman Test.

## Data Availability

The datasets generated during and/or analysed during the current study are available from the first or corresponding author on reasonable request.
